# Down-regulation of ABCG2 and ABCB4 transporters in the placenta of rats exposed to cadmium

**DOI:** 10.18632/oncotarget.9415

**Published:** 2016-05-17

**Authors:** Lili Liu, Liang Zhou, Shuiwang Hu, Shanyu Zhou, Yingyu Deng, Ming Dong, Jianxun Huang, Yuli Zeng, Xiaoyan Chen, Na Zhao, Hongling Li, Zhenhua Ding

**Affiliations:** ^1^ Department of Radiation Medicine, School of Public Health and Tropic Medicine, Southern Medical University, Guangzhou, China; ^2^ Department of Toxicology, Guangdong Province Hospital for Occupational Disease Prevention and Treatment, Guangzhou, China; ^3^ Department of Pathophysiology, Southern Medical University, Guangzhou, China

**Keywords:** cadmium, proteomics, placenta, ABCG2, ABCB4

## Abstract

As a maternal and developmental toxicant, cadmium (Cd) possesses weak penetrability through the placental barrier. However, the underlying mechanism remains unclear. To gain insight into the protein molecules associated with Cd toxicity in placenta and explore their roles in Cd transportation, a reproductive animal experiment was carried out using Sprague-Dawley rats. We performed proteomic analysis of the placenta by Difference Gel Electrophoresis (DIGE) combined with Matrix-Assisted Laser Desorption/Ionization Time-of-Flight Tandem Mass Spectroscopy (MALDI-TOF/TOF MS). The DIGE assay identified 15 protein spots that were differentially expressed with a greater than 1.5-fold change in placenta of Cd-treated rats compared to the control rats. Based on the expression patterns and biological functions of the proteins, we selected the ABCG2 and ABCB4 transporter proteins for further analysis. Western blot analysis showed that Cd exposure could down-regulate the expression of ABCG2 and ABCB4 in the placenta. There was a negative dose-response relationship between Cd exposure and the expression of ABCG2 or ABCB4 protein. These results indicated that down-regulation of ABCG2 and ABCB4 transporters may regulate Cd across through placenta and thus affect the *in vivo* toxic effect of Cd to fetus.

## INTRODUCTION

Cadmium (Cd) is extensively used in industry and widespread in the environment. Cd is absorbed into human body through food, drink, inhalation and smoking. In addition to kidney, liver and bone, placenta is the main target organ where Cd accumulates [1]. One of the major sources of Cd hazard is occupational exposure. As most of the occupational workers are young adults, the reproductive toxicity among the female employees and the adverse effects in their offsprings become the concern of public health and occupational medicine. Many studies on the reproductive toxicity of Cd exposure used experimental models [2, 3] and rarely examined the long term toxic effects of Cd. Since acute toxic effect of Cd exposure is distinctly different from that of chronic damage, the reproductive toxicity of low-dose and long term Cd exposure remains obscure.

The placenta is the interface for nutrition and gas exchange between the mother and fetus. It is the organ that could be used to assessing maternal and fetal health affected by Cd exposure [4]. There are some evidences show that Cd concentration in human fetal cord blood accounts for only 10% of the concentration in maternal blood [5, 6]. This indicates that the placenta has a barrier effect against Cd penetration [6–8]. However, little is known about the underlying molecular mechanism due to the lack of knowledge in proteomic change in response to Cd exposure in placenta.

Cd possesses higher affinity with the sulfhydryl (thiol) groups of albumin and metallothionein (MT) [9], which results in MT functioning as a sequestering molecule for a large fraction of tissue Cd [10] and likely to be responsible for placental storage of heavy metals [11, 12]. However, MT can also nonspecifically bind to some divalent metals, e.g., lead (Pb) and mercury (Hg), to decrease their toxicity in the placenta [13–15]. One recent study [16] proposed that MT in the uterus and placenta does not play a significant role in preventing Cd transport from the uterus through the placenta to the fetus.

ATP-binding cassette (ABC) transporters represent one of the largest families of membrane proteins from prokaryotes to humans, which couple the energy derived from ATP hydrolysis essentially to translocate, among various substrates, toxic compounds including heavy metals across the cell membrane [17, 18]. Among the ABC transporters, ABCC1 and ABCG2 are known to be active pumps for heavy metals. ABCC1 (MRP1) is ubiquitously expressed in normal tissues and is an active pump of glutathione, glucuronate and sulfate conjugated and unconjugated organic anions of toxicological relevance. Substrates of MRP1 include lipid peroxidation products, herbicides, tobacco, specific nitrosamines, mycotoxins, heavy metals, natural product and antifolate anti-cancer agents [19]. Heavy metals such as cadmium, lead and mercury are known to be neurotoxic to developing fetus, and ABCG2 which is an efflux transporter located in the maternal facing membranes of human placenta protects fetus from xenobiotics by transferring compounds from syncytiotrophoblast to maternal circulation [20]. Therefore, we speculated that some proteins other than MT might play a direct role in regulating Cd toxicity in placenta.

To probe the protein molecules that regulate the toxicity of low level and long term Cd exposure in placenta, we carried out a consecutive 44-day Cd exposure experiment to mimic occupational conditions in Sprague-Dawley (SD) female rats. We tested whether advanced mass-spectrometry-based proteomics could reveal the potential targets that regulating low level Cd exposure in the rat placenta. For this purpose, we analyzed the proteomic profiles of the placenta using Difference Gel Electrophoresis (DIGE) combined with Matrix-Assisted Laser Desorption/Ionization Time-of-Flight Tandem Mass Spectroscopy (MALDI-TOF/TOF MS). Differentially expressed proteins were screened and further validated by Western blot analysis. The pathological change of placenta was analyzed.

## RESULTS

### The response of parental females and fetuses to Cd exposure

**Figure 1:**
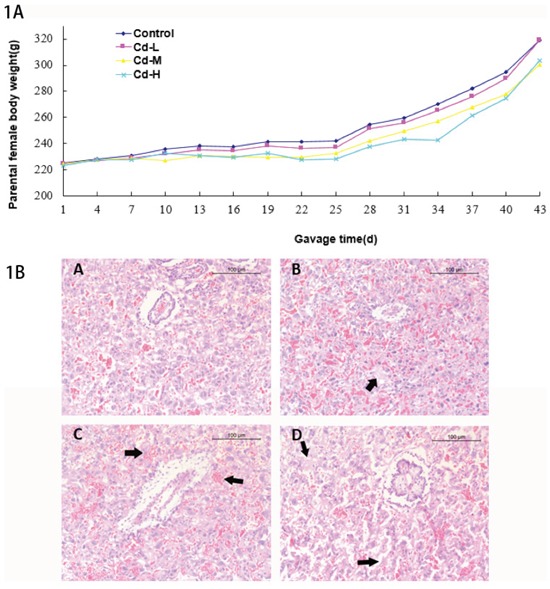
**A.** Body weights of parental females. **B.** The histological analysis of placentas in different treatment groups. (A): control group. B: Cd-L group. Mild degradation of cytotrophoblastic cells was seen (arrow). C: Cd-M group. Placental hyperemia and decreased glycogen cells were observed (arrow). D: Cd-H group. Dramatic degradation of cytotrophoblastic cells was seen (arrow).

All animals looked normal except that the female rats showed slow body-weight gain from gavage day 19 in the Cd-M and Cd-H groups compared with the control group; and the growth difference had a statistical significance (*P*<0.05). Although body-weight gain in Cd-L group was also slow, there was no statistical significance compared with the control group (Figure 1A). These results suggested that Cd exposure slowed the body growth of the female rats.

The histological analysis on placentas showed mild degradation of cytotrophoblastic cells in the Cd-L group. In the Cd-M and Cd-H groups, placental hyperemia, decreased glycogen and degradation of cytotrophoblastic cells were observed (Figure 1B).

Compared with that of the control group, there was no significant change in the pregnant frequency and litter size in the Cd-L group, neither difference of developmental toxicity to fetuses was found on gestation day 20 as shown in detail in Table 1.

**Table 1:** Maternal toxic response and developmental endpoints on gestation day 20

Group	Control	Cd-L
No. of pregnancy	7	8
No. of litter size (mean±S.D.)	10.71±2.93	10.00±3.46
No. of corpora lutea	115	131
No. of implantation	99	104
No. (percentage) of live foetuses	75 (75.76%)	80 (76.92%)
No. (percentage) of dead foetuses	2 (2.02%)	0 (0)
No. (percentage) of resorption	22 (22.22%)	24 (23.08%)

The above results demonstrated that exposure to low level of Cd had no toxic effect to parental females and fetuses.

### Cd concentrations in parental and umbilical cord blood

**Figure 2:**
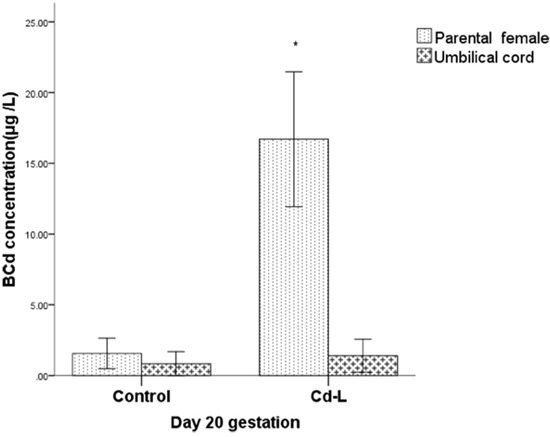
Cd concentrations in parental and umbilical cord blood * indicates significantly different from the control group, P<0.05.

The Cd blood concentration in the Cd-L group was significantly increased in the female rats compared with the control group on gestation day 20 (*P*<0.05). However, the Cd concentration in the umbilical cord blood remained equivalent with that of the control group (*P*>0.05) (Figure 2). These results indicated that low level of Cd did not penetrate through the placenta, although the placenta was one of the main target organs exposed to Cd.

### Proteomic analysis identifies the differential expression of ABCG2 and ABCB4 in placenta

**Figure 3:**
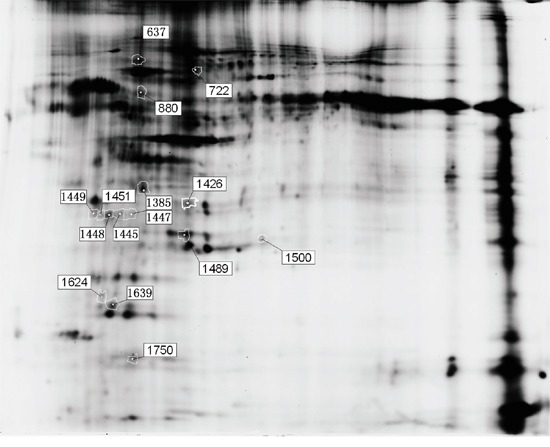
Fifteen differentially expressed protein spots in the two dimensional-fluorescence difference gel electrophoresis (DIGE) Proteins were extracted from placentas of female SD rats as described in the text. The first dimension was pH 3–10 NL IPG strips, and the second dimension was 12.5% polyacrylamide. The image of fluorescently labeled proteins was acquired using a Typhoon 9400 scanner.

**Figure 4:**
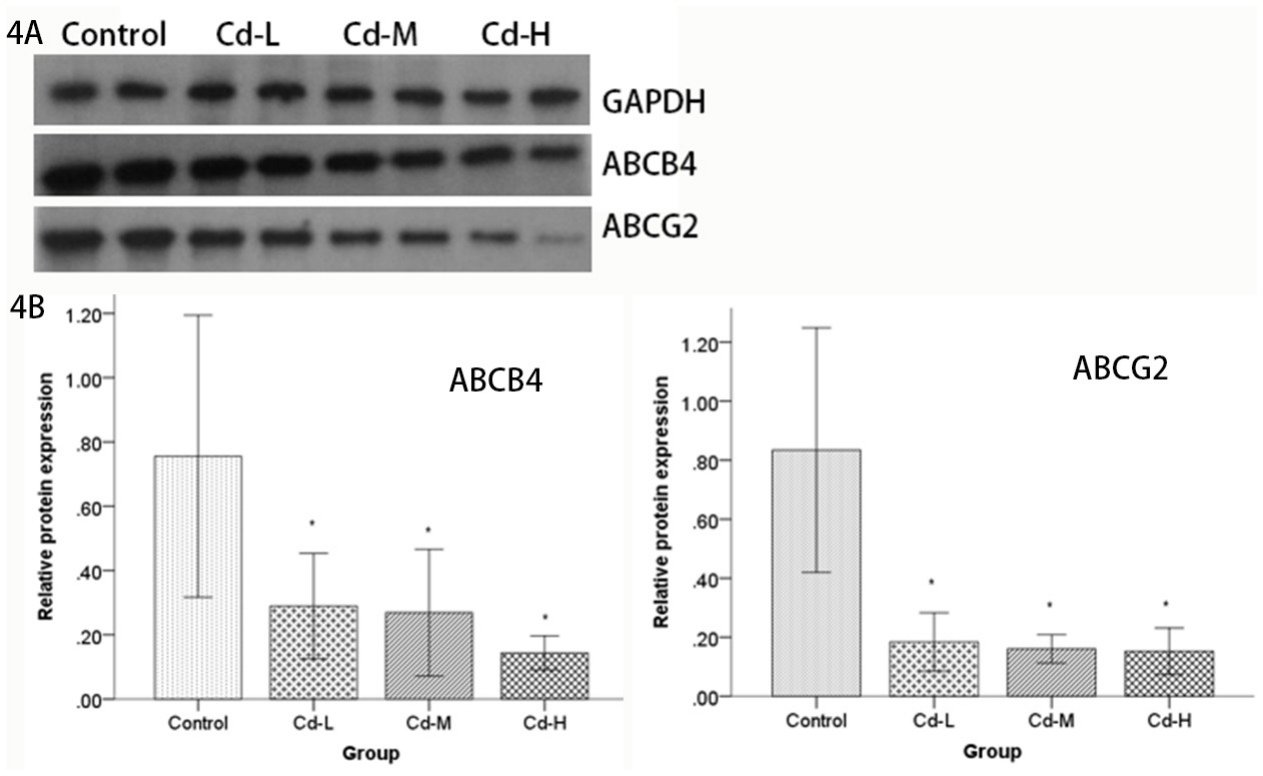
Analysis of ABCB4 and ABCG2 protein expression by Western blot **A.** Cell lysates from the placentas of female SD rats were electrophoretically separated by SDS-PAGE, transferred to PVDF membranes and probed using ABCB4 and ABCG2 antibodies. ABCB4 and ABCG2 levels were compared between the Cd-treated groups and the control group. **B.** Relative quantities based on OD are shown. Data are presented as means±SD of relative fold changes. *P<0.05, compared with the control group (one-way ANOVA).

To explore the reason underlying the difference of Cd distribution in the parental and umbilical cord blood, we performed proteomic analysis to screen for differentially-expressed molecules that are in the placenta. According to the screening criteria, we identified 15 protein spots with a greater than 1.5-fold change relative to the controls in Cd-L and Cd-H groups. The positions of these spots are indicated in Figure 3. Uniprot database analyses identified the protein spots and their variations, which are listed in Table 2. Some of the proteins were repeatedly detected, such as C-reactive protein and Apolipoprotein. Based on the expression patterns and biological functions of the identified protein spots in each group, it seemed that multidrug resistance 3 (MDR3/ABCB4) and ABCG2/BCRP transporter proteins were the most prominent. Both ABCG2 and ABCB4 belong to the ATP-binding cassette (ABC) transporter sub-family that affects the absorption, distribution and excretion of drugs and environmental toxins. To validate the proteomics results, the expression of ABCG2 and ABCB4 were further examined by Western blot using specific, commercially available antibodies. As shown in Figure 4A, the levels of ABCG2 and ABCB4 expression were negatively correlated with Cd exposure, being significantly down-regulated in the treated groups (*P*<0.05). Interestingly, we found a dose-response relationship between Cd exposure and ABCG2 or ABCB4 protein expression (Figure 4B). It could be speculated that ABCG2 and/or ABCB4 might be responsible for the removal of Cd out of the placenta.

**Table 2:** Proteins identified by mass spectrometry

Spot No.	Access. No.	Protein name	Ion Score	Ion Score (C.I.%)	PI	MW (KD)	Ratio	
							Cd-L/N	Cd-H/N
637	Q08201	ATP-binding cassette sub-family B member 4	20	64.724	9.11	141.1	−1.52	−1.06
722	P06761	78 Kda glucose-regulated protein	67	99.858	5.07	72.5	1.09	1.48
880	Q6P502	T-complex protein 1 subunit gamma	43	61.782	6.23	61.2	−1.47	−1.40
1385	P02650	Apolipoprotein E	86	99.998	5.23	35.8	−1.46	−1.19
1426	P67779	Prohibitin	234	100	5.57	29.9	1.01	1.47
1445	P41738	Aryl hydrocarbon receptor	27	92.505	6.04	97.1	−1.51	−1.15
1447	P48199	C-creactive protein	59	99.04	4.89	25.7	−1.45	−1.13
1448	P48199	C-creactive protein	136	100	4.89	25.7	−1.46	−1.29
1449	P48199	C-creactive protein	49	87.143	4.89	25.7	−1.46	−1.14
1451	P48199	C-creactive protein	95	100	4.89	25.7	−1.33	−1.52
1489	P04639	Apolipoprotein A-1	151	100	5.52	30.1	−1.49	1.12
1500	Q80W57	ATP-binding cassette sub-family G member 2	25	87.234	9.21	73.4	1.48	−1.04
1624	P02680	Fibrinogen gamma chain	57	98.547	5.62	51.2	−1.51	1.44
1639	P13832	Myosin regulatory light chain RLC-A	70	99.927	4.67	19.9	−1.51	1.05
1750	P11762	Galectin-1	191	100	5.14	15.2	1.45	1.62

N: control group. Ratio: the ratio of proteins from the Cd-L and Cd-H group to proteins from the control group.

M: molecular weight. pI: isoelectric point.

## DISCUSSION

Most studies on chronic animal toxicity and carcinogenicity of Cd used doses in the range of 1-5 mg/kg body weight/day [21, 22]. In Japan, young female Wistar rats were exposed to 1 mg Cd/L in drinking water for 24 months to simulate a low level lifetime exposure to Cd [23]. Some studies determined that an orally administered dose of 1 mg/kg body weight/day to rats represented a level similar to the daily Cd intake in humans [16]. The World Health Organization established a Provisional Tolerable Weekly Intake (PTWI) of dietary Cd intake of 7 μg/kg body weight/day in humans [24]. As the daily intake of Cd from the intestinal tract in experimental rats was deduced to be approximately 0.36-0.54% of oral Cd administration [25], we used 1 mg/kg body weight/day as a low level of exposure of Cd in animals to mimic the common levels of Cd exposure. The actual intake of Cd into the body was estimated to be slightly lower than that of the PTWI.

In the present study, the mean concentration of Cd in the urine of Cd-L rats on Day 20 of gestation was 5.48 (4.03~9.23) μg/L which belongs to low level exposure according to *Diagnostic Criteria of Occupational Cadmium Poisoning* in China [26]. We found that the blood concentration of Cd-L rats was significantly higher than the controls. However, Cd concentration in umbilical cord blood had no difference with the controls. These results indicated that Cd did not enter into umbilical cord blood from placenta in rats with low level of Cd exposure.

The above results demonstrated that placenta may play a role in preventing Cd transportation from mother to fetus. This result inspired us to investigate the mechanisms responsible for the regulation of the absorption and excretion of Cd during pregnancy. To gain insight into the proteins associated with Cd exposure, the placental protein molecules were analyzed by DIGE Proteomic analysis, and further validated by Western blot analysis, revealed that the removal of Cd may associate with the expression of ABCG2 and ABCB4 proteins that belong to the members of ABC transporter sub-family.

ABC transporters are one of the largest membrane protein super-families and are present in all species from prokaryotes to mammals [17, 18]. They have ATP-binding sites which play a fundamental role in the transportation of substrates out of cells or into subcellular compartments against gradient fueled by ATP hydrolysis [27, 28]. ABCG2, also named the breast cancer resistance protein (BCRP), was a drug efflux transmembrane protein [29]. ABCG2 is also called placenta-specific ATP-binding cassette gene (ABCP) [30], It is expressed in a variety of organs including the brain, liver, kidney, small intestine, bone marrow and placenta [31–34]. ABCG2 mediates the disposition and excretion of numerous endogenous chemicals and xenobiotics [35]. There was evidence suggests that ABCG2 is involved in mediating the toxicity of Cd although its mRNA expression was not affected *in vitro* [20]. Surprisingly, we found that the expression of ABCG2 in placenta had a negative dose-dependent relationship with Cd concentration. We propose that the down-regulation of ABCG2 is a negative factor of protective mechanisms in response to maternal Cd exposure to prevent the permeabilization of Cd across the placenta and thus affect the *in vivo* toxic effect of Cd to fetus. It is not clear why Cd down-regulates but not up-regulates the ABCG2 protein. A recent study on rats using 6 mg/kg Cd exposure for 4 weeks demonstrated down-regulation of OAT1, MRP4, and ABCG2 in the kidney [36], which could support our finding.

Although there was a difference between Proteomic analysis and Western blot validation in ABCG2 transporter, it could result from protein modification leading to changes in protein expression. Cd is a ligand for the antioxidant nuclear factor erythroid 2-related factor 2 (Nrf-2) which is a regulator of ABCG2 expression [37, 38]. It was suggested that inhibition of ABCG2 may be due to the interference with ATP-synthesis in cells through reactive oxygen species induced by Cd [39].

As a transporter, ABCB4 gene encodes the MDR3 protein which has a specific physiological function with intrahepatic cholestasis as a phosphatidylcholine carrier and transports only specific compounds [40–42]. Recently, mutations of ABCB4 were reported in early-onset gallstone disease, cholestasis of pregnancy, liver cirrhosis, and hepatobiliary cancer [43, 44]. Moreover, multiple transportation functions of ABCB4 were discovered such that it could be a multixenobiotic transporter and active barrier against chemical uptake in zebrafish embryos [45]. It was also reported that ABCB4 can bind to the transcription factor HNF4 α through the promoter site regulating drug transport across the blood-brain barrier [46]. Furthermore, our analysis demonstrated that the level of ABCB4 expression was significantly down-regulated by Cd exposure similar to ABCG2. The above results suggest that ABCB4 and ABCG2 transporters may act some roles in minimizing the toxic effects of Cd to the fetus. Therefore, further experiments are needed to understand whether ABCB4 and ABCG2 could act as transporters in placental barrier especially for Cd.

In summary, our study is the first to provide evidence that Cd exposure causes down-regulation of ABCG2 and ABCB4 in rat placenta. The down-regulation of ABCG2 and ABCB4 transporters during maternal exposure to Cd may be a critical mechanism responsible for Cd toxicity to fetus. Future studies will focus on the mechanism of down-regulation of the two transporters associated with Cd exposure in placenta.

## MATERIALS AND METHODS

### Animals

This study was carried out in strict conformity with the recommendations in the Guide for the Care and Use of Laboratory Animals of the National Institutes of Health. All animal experiments were conducted with the approval of the Animal Ethical Committee of *Guangdong Province Hospital for Occupational Disease Prevention and Treatment (GDHOD)* (Permit Number: 20130011). All surgery was performed under sodium pentobarbital anesthesia, and we made every effort to minimize the suffering of the animals.

Specific Pathogen-Free (SPF) 8-week-old SD rats were purchased from *Guangdong Medical Laboratory Animal Center* (GDMLAC, certified No. SCXK2013-0002) and housed in the SPF animal facility under controlled temperature (22±3°C), relative humidity (40-70%) and lighting (12-hr light/dark). All animals were fed in their respective cages with *ad libitum* access to food and ultrapure water, which was monitored to not exceed the standards for metals. After a 5-day acclimation period, female rats were randomly assigned to control and treatment groups (8 females per group). Low-dose Cd (Cd-L), median-dose Cd (Cd-M) and high-dose Cd (Cd-H) groups were treated with CdCl_2_ (Sigma-Aldrich, MO, USA) at 1, 3 and 9 mg/kg body weight/day, respectively. The doses were based on our previous study [47] and the literature reports [16, 25, 48]. All female rats were administered Cd by gavage for 20 consecutive days. The female rats were weighed every 3 days, and the dose was adjusted according to body weight. The control group was given only ultrapure water by gavage. After 20 days of gavage, the female rats were mated with the male rats, which were purchased at the same time. The day on which a vaginal plug was observed was assigned as gestation day 0 according to the OECD guideline [49]. The Cd-L and control groups were euthanized on gestation day 20, the Cd-M and Cd-H groups were allowed to deliver.

### Sample collection and Cd determination

On gestation day 20, blood samples from the Cd-L and control groups were collected via the aorta abdominalis before the parental females were euthanized. The uteri were quickly moved, the fetuses were separated and the umbilical cord blood was collected. The numbers of corpora luteas, implantations, live or dead fetuses, resorptions were recorded to represent the developmental toxicity endpoints according to the OECD guideline [50]. The placentas were harvested after washing with ice-cold saline solution. The excess liquid was absorbed with filter paper, and the tissues were frozen in liquid nitrogen and preserved at −80°C. Remaining tissues were fixed in neutral formalin. The placentas of the Cd-M and Cd-H groups were immediately collected after parturition and prepared for proteomic analysis. Separate surgical instruments were used for each group and were soaked in dilute nitric acid prior to surgery.

All of the blood samples were diluted 10-fold with matrix modifier and the concentration of Cd was determined using an atomic absorption spectrometer (model ZEEnit 700, AAS, Analytikjena Co., Germany).

### Placental protein extraction, purification and quantification

All reagents were supplied by the Sigma Chemical Company (Sigma-Aldrich, MO, USA) unless otherwise noted. To minimize the effects of differences among individual animals, 100 mg of each placental sample from the same treatment group were mixed together. After the placentas were cut into pieces and homogenized, the supernatants were discarded, and the samples were resuspended in lysis buffer (30 mM Tris-HCl, 7 M urea, 2 M thiourea, 4% w/v 3-[(3-cholamidopropy) dimethylammonio]-1 propanesulfonate (CHAPS), and a protease inhibitor mixture, pH 8.5). After incubating on ice for 1 h, the suspensions were sonicated 10 times (3-s bursts with 7-s pauses) on ice to prevent sample overheating. These lysates were centrifuged at 20,000 g for 1 h at 4°C. The suspended proteins were precipitated, cleaned using the 2-D Clean-up Kit (GE Healthcare) according to the manufacturer's instructions and resuspended in lysis buffer. The protein concentration was determined using the 2-D Quant Kit (GE Healthcare) in accordance with the manufacturer's protocol [51].

### DIGE analysis

DIGE analysis was performed as described previously with some modifications [51, 52]. A total of 50 μg of protein from each group was labeled with CyDye 3 (control group) or CyDye 5 (Cd-L group) in gel No. 1 and with CyDye 3 (Cd-H group) or CyDye 5 (control group) in gel No. 2. An internal standard (100 μg) consisting of equal amounts of proteins from all of the groups was labeled with CyDye 2 and divided between the two gels. The CyDyes were dissolved in anhydrous dimethylformamide and mixed with the samples at a ratio of 400 pmol of CyDye to 50 μg of protein. The reaction was conducted on ice for 30 min in the dark and was terminated by adding 1 μL of 10 mM lysine under the same conditions for 10 min. Each labeled sample was dissolved with rehydration buffer and separated with a 24-cm immobilized pH gradient gel strip [pH 3-10 NL] in the first dimension. First-dimension isoelectric focusing (IEF) was carried out at 20°C in an Ettan™ IPGphor (Sigma, MO, USA) using the following protocol: 30 V for 12 h, 100 V for 1 h, 1,000-V gradient for 1 h, 8,000-V gradient for 2 h, and then 8,000 V for 50,000 volt-h followed by a 500-V hold. The strips were equilibrated by agitation for 30 min, loaded onto a 12.5% SDS-polyacrylamide gel electrophoresis (SDS-PAGE) and run on an Ettan™ DALT Six System (GE Healthcare). After the 2-DE run, the gels were scanned with a Typhoon 9400 imager (GE Healthcare) and analyzed with DeCyder2D software v6.5 (GE Healthcare). Protein spots with a fold change ≥1.5 in all groups were chosen for further analysis.

### Protein identification

After imaging analysis, a preparative gel was run using 800 μg of pooled protein sample, stained with Coomassie blue G-250 and matched to the immunoblotting images. Selected spots were chosen automatically from the preparative gel using an Ettan Spot Picker (GE Healthcare). Identification of proteins was performed using the protocol described previously by our group [52]. The selected spots were destained with 30% acetonitrile (ACN)/100 mM NH_4_HCO_3_, dehydrated with 100% ACN for 5 min, and dried using a centrifugal concentrator (TOMY SEIKO, Tokyo). Then, 2 μL of 25 ng/μL trypsin (Promega) diluted in 50 mM NH_4_HCO_3_ was added to each gel piece and incubated for 30 min at 4°C, and 30 μl of 50 mM NH_4_HCO_3_ was added. The spots were incubated overnight at 37°C. Finally, 100 μL of 60% (v/v) ACN in 0.1% aqueous trifluoroacetic acid (TFA) was added to the gel pieces, which were then sonicated twice for 15 min. The combined solutions were collected and freeze-dried to 10 μL. The peptides obtained after in-gel trypsin digestion were purified with a ZipTip® C18 (Millipore), and the eluent was spotted onto a target plate of a MALDI linear reflectron mass spectrometer with a 1:1 mixture of 5 mg/mL of a-cyano-4-hydroxycinnamic acid (CHCA, Sigma, MO, USA) as the matrix. The digests were performed using an ABI 4800 MALDI-TOF/TOF MS (Applied Biosystems, Foster City, USA). MS peptide mass fingerprinting (PMF) was retrieved with Mascot software, whereas proteins were identified with MS/MS patterns. The molecular weight range and quality error tolerance were 800–4000 Da and 50 ppm, respectively. The MASCOT search engine (v2.1, Matrix Science) was used to search all of the tandem mass spectra. GPS Explorer™ software v3.6.2 (Applied Biosystems, Foster City, USA) was used to create and search files with the MASCOT search for peptide and protein identification. Protein identities were obtained by using the MASCOT search engine against the rat Swiss-Prot non-redundant sequence databases.

### Western blot

The differentially expressed proteins ABCB4 and ABCG2 were further validated using Western blot analysis as previously described [51]. GAPDH (Abcam, Cambridge, USA) was used as a loading control. Equal amounts of proteins (50 μg) were extracted from samples, electrophoretically separated by 12.5% SDS-PAGE, and transferred to a PVDF membrane. Subsequently, the membranes were blocked in TBST containing 5% skim milk for 1 h at room temperature and incubated with a rabbit polyclonal primary antibody against ABCB4 or ABCG2 (1:1000, Origene, Rockville, USA) for 2 h at room temperature, followed by incubation with a peroxidase-conjugated secondary antibody (diluted 1:8000, Abcam, Cambridge, USA) for 1 h at room temperature. After washing with TBST three times, the bound primary antibody was visualized with ECL Western blotting substrate kit (Forevergen Biosciences, Guangzhou, China). Then, images were captured using a Carestream Image Station 4000R (Carestream Health, Rochester, USA) and analyzed with Quantity One software (Bio-Rad, Hercules, USA).

### Histological analysis

Pathological changes of placentas were analyzed using standard histological procedures. Briefly, 4-μm-thick sections of formalin-fixed, paraffin-embedded placenta were deparaffinized and rehydrated with graded concentrations of alcohols, and then stained with hematoxylin and eosin (H&E). The histological analysis was performed according to the previous method [53].

### Statistical analysis

The statistical software package SPSS (v13.0) was used for statistical analysis. The data were expressed as means and standard deviations according to a normal distribution or as medians with interquartile ranges. Differences among experimental groups were examined by one-way ANOVA. *P*<0.05 was considered significant.

## References

[1] Zadorozhnaja TD, Little RE, Miller RK, Mendel NA, Taylor RJ, Presley BJ, Gladen BC (2000). Concentrations of arsenic, cadmium, copper, lead, mercury, and zinc in human placentas from two cities in Ukraine. J Toxicol Environ Health A.

[2] Wang Z, Wang H, Xu ZM, Ji YL, Chen YH, Zhang ZH, Zhang C, Meng XH, Zhao M, Xu DX (2012). Cadmium-induced teratogenicity: Association with ROS-mediated endoplasmic reticulum stress in placenta. Toxicol Appl Pharmacol.

[3] Cazan AM, Klerks PL (2015). Effects from a short-term exposure to copper or cadmium in gravid females of the livebearer fish (Gambusia affinis). Ecotoxicol Environ Saf.

[4] lyengar GV, Rapp A (2001). Human placenta as a ‘dual’ biomarker for monitoring fetal and maternal environment with special reference to potentially toxic trace elements. Part 3: toxic trace elements in placenta and placenta as a biomarker for these elements. Sci Total Environ.

[5] Osman K, Akesson A, Berglund M, Bremme K, Schütz A, Ask K, Vahter M (2000). Toxic and essential elements in placentas of Swedish women. Clin Biochem.

[6] Kantola M, Purkunen R, Kröger P, Tooming A, Juravskaja J, Pasanen M, Saarikoski S, Vartiainen T (2000). Accumulation of cadmium, zinc, and copper in maternal blood and developmental placental tissue: differences between Finland, Estonia, and St. Petersburg. Environ Res.

[7] Lin CM, Doyle P, Wang D, Hwang YH, Chen PC (2011). Does prenatal cadmium exposure affect fetal and child growth?. Occup Environ Med.

[8] Sakamoto M, Yasutake A, Domingo JL, Chan HM, Kubota M, Murata K (2013). Relationships between trace element concentrations in chorionic tissue of placenta and umbilical cord tissue: potential use as indicators for prenatal exposure. Environ Int.

[9] Nordberg G, Kjellstrom T, Nordberg M, Friberg L, Elinder C, Kjellstrom T, Nordberg G (1985). Chapter 6 Kinetics and Metabolism. Cadmium and Health: A Toxicological and Epidemiological Appraisal, Vol. 1. Exposure, Dose and Metabolism.

[10] Shaikh ZA (1982). Metallothionein as a storage protein for cadmium: its toxicological implications. Dev Toxicol Environ Sci.

[11] Goyer RA, Haust MD, Cherian MG (1992). Cellular localization of metallothionein in human term placenta. Placenta.

[12] Kippler M, Hoque AM, Raqib R, Ohrvik H, Ekström EC, Vahter M (2010). Accumulation of cadmium in human placenta interacts with the transport of micronutrients to the fetus. Toxicol Lett.

[13] Ma HY, Li H, Wang JC, Xu FS (2006). Expression and significance of metallothionein in the placenta of women with low level lead exposure during pregnancy. [Article in Chinese]. Zhonghua Fu Chan Ke Za Zhi.

[14] Shimada A, Yamamoto E, Morita T, Yoshida M, Suzuki JS, Satoh M, Tohyama C (2004). Ultrastructural demonstration of mercury granules in the placenta of metallothionein-null pregnant mice after exposure to mercury vapor. Toxicol Pathol.

[15] Benitez MA, Mendez-Armenta M, Montes S, Rembao D, Sanin LH, Rios C (2009). Mother-fetus transference of lead and cadmium in rats: involvement of metallothionein. Histol Histopathol.

[16] Nakamura Y, Ohba K, Suzuki K, Ohta H (2012). Health effects of low-level cadmium intake and the role of metallothionein on cadmium transport from mother rats to fetus. J Toxicol Sci.

[17] Kathawala RJ, Gupta P, Ashby CR (2015). Chen ZS. The modulation of ABC transporter-mediated multidrug resistance in cancer: a review of the past decade. Drug Resist Updat.

[18] Li W, Sharma M, Kaur P (2014). The DrrAB efflux system of Streptomyces peucetius is a multidrug transporter of broad substrate specificity. J Biol Chem.

[19] Leslie EM, Deeley RG, Cole SP (2001). Toxicological relevance of the multidrug resistance protein 1, MRP1 (ABCC1) and related transporters. Toxicology.

[20] Kummu M, Sieppi E, Wallin K, Rautio A, Vähäkangas K, Myllynen P (2012). Cadmium inhibits ABCG2 transporter function in BeWo choriocarcinoma cells and MCF-7 cells overexpressing ABCG2. Placenta.

[21] Beryllium, cadmium, mercury, and exposures in the glass manufacturing industry (1993). IARC Monogr Eval Carcinog Risks Hum.

[22] Waalkes MP (2000). Cadmium carcinogenesis in review. J Inorg Biochem.

[23] Brzóska MM, Moniuszko-Jakoniuk J (2004). Low-level lifetime exposure to cadmium decreases skeletal mineralization and enhances bone loss in aged rats. Bone.

[24] Food and Agriculture Organization/World Health Organization Expert Committee on Food Additives (2001). Evaluation of certain food additives and contaminants. World Health Organ Tech Rep Ser.

[25] Ohta H, Yamauchi Y, Nakakita M, Tanaka H, Asami S, Seki Y, Yoshikawa H (2000). Relationship between renal dysfunction and bone metabolism disorder in male rats after long-term oral quantitative cadmium administration. Ind Health.

[26] Ministry of Health of the People's Republic of China (2002). GBZ 17-2002 Diagnostic Criteria of Occupational Cadmium Poisoning.

[27] Endicott JA, Ling V (1989). The biochemistry of P-glycoprotein-mediated multidrug resistance. Annu Rev Biochem.

[28] Xiao J, Wang Q, Bircsak KM, Wen X, Aleksunes LM (2015). In Vitro Screening of Environmental Chemicals Identifies Zearalenone as a Novel Substrate of the Placental BCRP/ABCG2 Transporter. Toxicol Res(Camb).

[29] Doyle LA, Yang W, Abruzzo LV, Krogmann T, Gao Y, Rishi AK, Ross DD (1998). A multidrug resistance transporter from human MCF-7 breast cancer cells. Proc Natl Acad Sci U S A.

[30] Allikmets R, Schriml LM, Hutchinson A, Romano-Spica V, Dean M (1998). A human placenta-specific ATP-binding cassette gene (ABCP) on chromosome 4q22 that is involved in multidrug resistance. Cancer Res.

[31] Ishikawa T, Nakagawa H (2009). Human ABC transporter ABCG2 in cancer chemotherapy and pharmacogenomics. J Exp Ther Oncol.

[32] Noguchi K, Katayama K, Mitsuhashi J, Sugimoto Y (2009). Functions of the breast cancer resistance protein (BCRP/ABCG2) in chemotherapy. Adv Drug Deliv Rev.

[33] Evseenko DA, Paxton JW, Keelan JA (2007). Independent regulation of apical and basolateral drug transporter expression and function in placental trophoblasts by cytokines, steroids, and growth factors. Drug Metab Dispos.

[34] Myllynen P, Kummu M, Sieppi E (2010). ABCB1 and ABCG2 expression in the placenta and fetus: an interspecies comparison. Expert Opin Drug Metab Toxicol.

[35] Bircsak KM, Aleksunes LM (2015). Interaction of Isoflavones with the BCRP/ABCG2 Drug Transporter. Curr Drug Metab.

[36] Wang J, Pan Y, Hong Y, Zhang QY, Wang XN, Kong LD (2012). Quercetin Protects against Cadmium-Induced Renal Uric Acid Transport System Alteration and Lipid Metabolism Disorder in Rats. Evid Based Complement Alternat Med.

[37] Chen J, Shaikh ZA (2009). Activation of Nrf2 by cadmium and its role in protection against cadmium-induced apoptosis in rat kidney cells. Toxicol Appl Pharmacol.

[38] Hagiya Y, Adachi T, Ogura S, An R, Tamura A, Nakagawa H, Okura I, Mochizuki T, Ishikawa T (2008). Nrf2-dependent induction of human ABC transporter ABCG2 and heme oxygenase-1 in HepG2 cells by photoactivation of porphyrins: biochemical implications for cancer cell response to photodynamic therapy. J Exp Ther Oncol.

[39] Liu J, Qu W, Kadiiska MB (2009). Role of oxidative stress in cadmium toxicity and carcinogenesis. Toxicol Appl Pharmacol.

[40] de Vree JM, Jacquemin E, Sturm E, Cresteil D, Bosma PJ, Aten J, Deleuze JF, Desrochers M, Burdelski M, Bernard O, Oude Elferink RP, Hadchouel M (1998). Mutations in the MDR3 gene cause progressive familial intrahepatic cholestasis. Proc Natl Acad Sci U S A.

[41] Van Helvoort A, Smith AJ, Sprong H, Fritzsche I, Schinkel AH, Borst P, van Meer G (1996). MDR1 P-glycoprotein is a lipid translocase of broad specificity, while MDR3 P-glycoprotein specifically translocates phosphatidylcholine. Cell.

[42] Smith AJ, van Helvoort A, van Meer G, Szabo K, Welker E, Szakacs G, Varadi A, Sarkadi B, Borst P (2000). MDR3 P-glycoprotein, a phosphatidylcholine translocase, transports several cytotoxic drugs and directly interacts with drugs as judged by interference with nucleotide trapping. J Biol Chem.

[43] Degiorgio D, Crosignani A, Colombo C, Bordo D, Zuin M, Vassallo E, Syrén ML, Coviello DA, Battezzati PM (2016). ABCB4 mutations in adult patients with cholestatic liver disease: impact and phenotypic expression. J Gastroenterol.

[44] Lammert F, Hochrath K (2015). A letter on ABCB4 from Iceland: On the highway to liver disease. Clin Res Hepatol Gastroenterol.

[45] Fischer S, Klüver N, Burkhardt-Medicke K, Pietsch M, Schmidt AM, Wellner P, Schirmer K, Luckenbach T (2013). Abcb4 acts as multixenobiotic transporter and active barrier against chemical uptake in zebrafish (Danio rerio) embryos. BMC Biol.

[46] Niehof M, Borlak J (2009). Expression of HNF4alpha in the human and rat choroid plexus – Implications for drug transport across the blood-cerebrospinal-fluid (CSF) barrier. BMC Mol Biol.

[47] Liu LL, Zhou ZY, Zhang AH (2012). The influence of enrichment stable isotope cadmium-112 exposure on development of fetal rats. [Article in Chinese]. Zhonghua Lao Dong Wei Sheng Zhi Ye Bing Za Zhi.

[48] Yoshida M, Ohta H, Yamaguchi Y, Seki Y, Sagi M, Yamazaki K, Sumi Y (1998). Age-dependent changes in metallothionein levels in liver and kidney of the Japanese. Biol Trace Elem Res.

[49] Test No (1983). 415: One-Generation Reproduction Toxicity Study. OECD Guidelines for the Testing of Chemicals.

[50] Test No (2001). 414: Prenatal Development Toxicity Study. OECD Guidelines for the Testing of Chemicals.

[51] Weng H, Liu F, Hu S, Li L, Wang Y (2014). GnRH agonists induce endometrial epithelial cell apoptosis via GRP78 down-regulation. J Transl Med.

[52] Tan Y, Liu TR, Hu SW, Tian D, Li C, Zhong JK, Sun HG, Luo TT, Lai WY, Guo ZG (2014). Acute coronary syndrome remodels the protein cargo and functions of high-density lipoprotein subfractions. PLoS One.

[53] Wang B, Wang S, Shao C, Wang G, Li Y, Cai L (2013). Proteomic characterization of the late and persistent effects of cadmium at low doses on the rat liver. J Appl Toxicol.

